# Extrafollicular Variant of the Common Adenomatoid Odontogenic Tumour: A Rare Case Report

**DOI:** 10.7759/cureus.87846

**Published:** 2025-07-13

**Authors:** Bhavani N Sangala, Kirti Buva, Saudamini More, Amit Patil, Sandhya Rani Akula, Sanpreet S Sachdev

**Affiliations:** 1 Oral Pathology and Microbiology, Bharati Vidyapeeth (Deemed to be University) Dental College and Hospital, Navi Mumbai, Mumbai, IND; 2 Public Health Dentistry, Bharati Vidyapeeth (Deemed to be University) Dental College and Hospital, Navi Mumbai, Mumbai, IND; 3 Conservative Dentistry and Endodontics, Bharati Vidyapeeth (Deemed to be University) Dental College and Hospital, Navi Mumbai, Mumbai, IND; 4 Oral Pathology and Microbiology, Kamineni Institute of Dental Sciences, Sreepuram, IND

**Keywords:** adenomatoid odontogenic tumour, anterior maxilla, extrafollicular variant, odontogenic neoplasm, root resorption

## Abstract

Adenomatoid odontogenic tumour (AOT) is a benign epithelial neoplasm of odontogenic origin, typically seen in young female patients and commonly associated with impacted canines in the anterior maxilla. The extrafollicular variant, which is not associated with unerupted teeth, is relatively rare and may present diagnostic challenges due to its similarity with other odontogenic cysts or tumours. This report presents a case of a 23-year-old female patient with a gradually enlarging swelling in the anterior maxilla. Radiographic imaging revealed a well-defined radiolucency with root resorption and displacement of adjacent teeth. Histopathological examination confirmed the diagnosis of extrafollicular AOT. The lesion was managed successfully with conservative surgical excision, and no recurrence was noted during a two-year follow-up. This case highlights the importance of integrating clinical, radiological, and histopathological findings for accurate diagnosis and appropriate management of this rare AOT variant.

## Introduction

Adenomatoid odontogenic tumour (AOT) is a relatively uncommon, benign epithelial lesion of odontogenic origin that demonstrates slow but progressive growth. It accounts for approximately 2.2% to 13% of all odontogenic tumours, depending upon the geographic location, placing it as the fourth most common entity within this group [1,2]. First described by Steensland in 1905 as “epithelial adamantinoma,” the lesion was later named “adenomatoid odontogenic tumour” by Philipsen and Birn in 1969 [1]. AOT is often referred to as the “two-thirds tumour” because two-thirds of the cases occur in female patients, two-thirds involve the maxilla, two-thirds are associated with unerupted teeth, and two-thirds of those are canines [3-5]. It predominantly affects adolescents and young adults, typically in the second decade of life, with a marked female predilection [2,3].

Histologically, AOT is characterized by proliferative odontogenic epithelium composed of spindle-shaped or polygonal cells arranged in characteristic rosettes, duct-like structures, and whorls, often embedded in a sparse connective tissue stroma that may exhibit calcifications and eosinophilic material [1,2]. Despite its histologic complexity, AOT maintains a benign behaviour and rarely recurs following conservative surgical excision [6]. Based on its clinical and radiographic presentation, AOT is classified into three variants: follicular (73%), extrafollicular (24%), and peripheral (3%) [7]. The follicular type is typically associated with an unerupted tooth, whereas the extrafollicular variant lacks this relationship, instead presenting within interradicular, periapical, or superimposed positions [7]. This classification plays a vital role in differential diagnosis, particularly because AOT can mimic more aggressive or cystic lesions both radiographically and clinically.

Given its variable presentations and the diagnostic challenges posed by its extrafollicular form, especially in atypical locations or age groups, detailed case documentation remains essential. The present report aims to contribute to the existing literature by highlighting the diagnostic features and clinical relevance of the rare extrafollicular variant of AOT.

## Case presentation

Clinical presentation

A 23-year-old female patient presented to the outpatient department with a chief complaint of swelling in the upper front region of the jaw, which had been gradually increasing in size over the past six months. The swelling was initially small and progressed steadily to reach its current dimensions. Extraoral examination revealed a solitary, diffuse swelling measuring approximately 5 × 5 cm located on the left middle third of the face. The swelling extended anteriorly from the philtrum of the upper lip to a perpendicular line from the outer canthus of the eye posteriorly, and from the infraorbital margin superiorly to about 2 cm below the ala-tragus line inferiorly. Obliteration of the nasolabial fold was observed.

**Figure 1:**
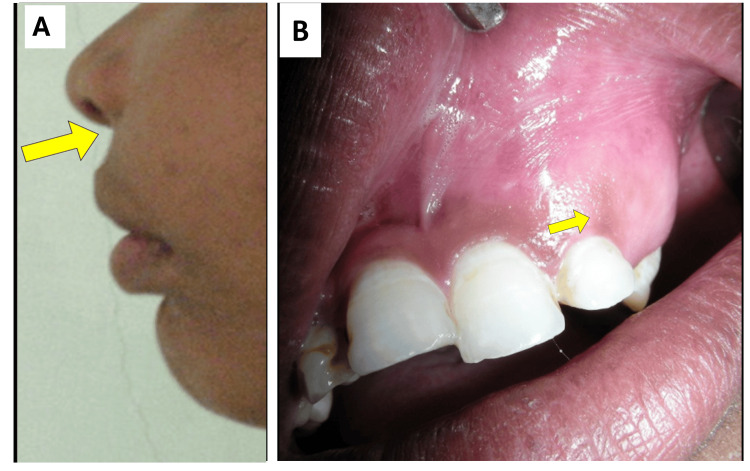
Clinical appearance of the swelling A) Swelling (indicated by the arrow) in the middle third of the face; B) Intraoral swelling in the maxillary anterior region (lesion indicated by the arrow).

Intraoral examination revealed a solitary swelling measuring approximately 4 × 2 cm in the left anterior maxillary region. The lesion extended from the midline anteriorly to the distal aspect of the left maxillary second premolar posteriorly, reaching superiorly into the maxillary labial vestibule and inferiorly up to the marginal gingiva. The swelling caused obliteration of the left upper buccal vestibular sulcus and expansion of both the buccal and palatal cortical plates. On palpation, the swelling was firm to hard in consistency. The clinical appearance of the lesion is shown in Figure 1.

Radiographic findings

**Figure 2:**
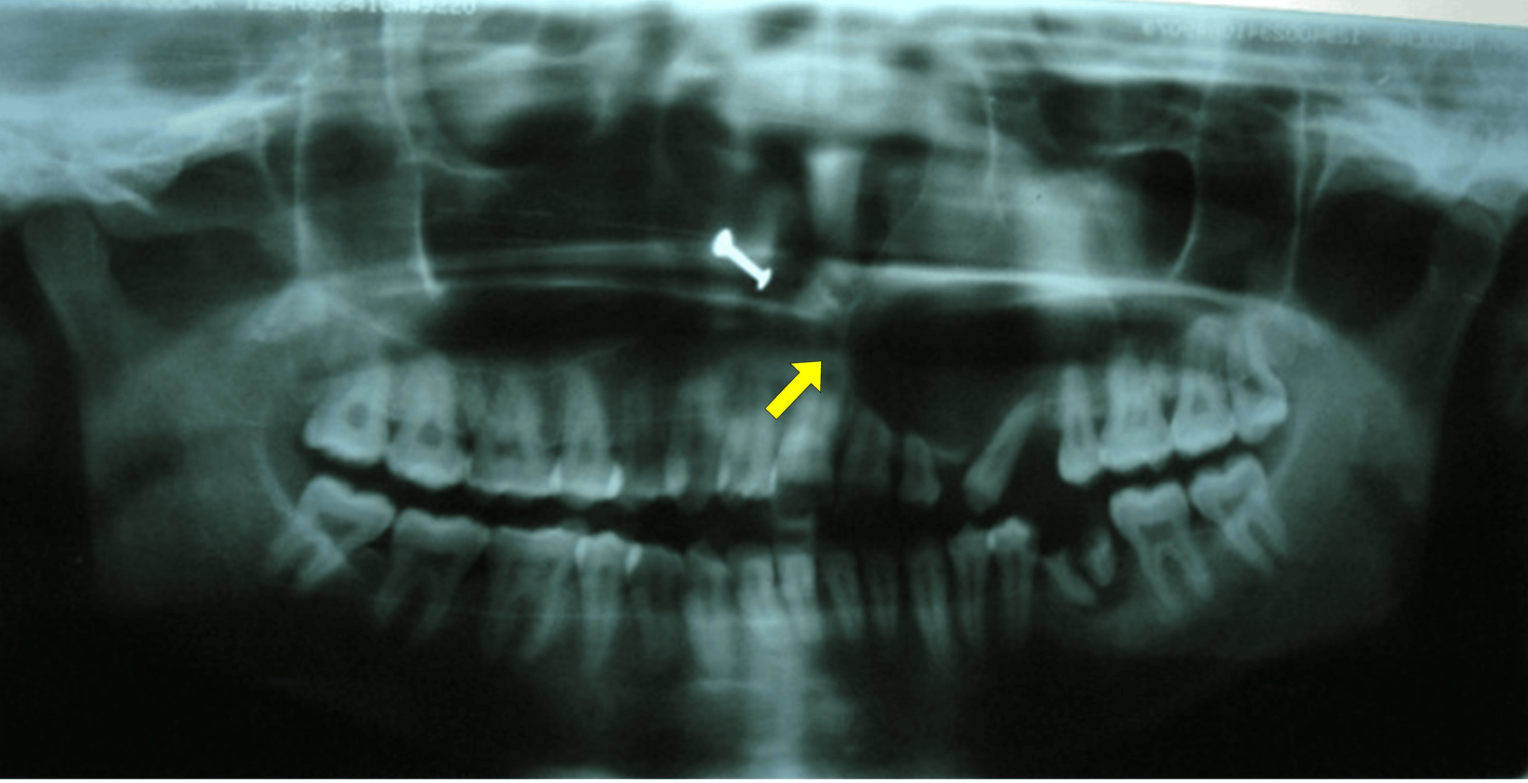
Orthopantomogram showing solitary well-defined radiolucency with a cortical margin (lesion indicated by the arrow)

A maxillary occlusal radiograph demonstrated a well-defined unilocular radiolucency with corticated margins extending from the midline anteriorly to the distal surface of the left maxillary first molar posteriorly. There was significant buccal cortical plate expansion and deviation of the nasal septum toward the right side. The nasal floor was intact. The left maxillary first premolar was missing as it was extracted 10 days ago, and the left maxillary canine was displaced mesially. An orthopantomogram (OPG) showed a solitary, well-defined unilocular radiolucency bordered by a thin radiopaque margin, measuring approximately 4 × 4 cm (Figure 2).

The lesion extended from the mesial surface of the left maxillary central incisor anteriorly to the mesial surface of the left maxillary first molar posteriorly. Vertically, it extended from the infraorbital margin superiorly to the cervical third of the adjacent teeth inferiorly. There was no involvement of the maxillary sinus or the nasal cavity. Evidence of root resorption was noted in the left maxillary central incisor, lateral incisor, and second premolar. 

Surgical management and gross examination

**Figure 3:**
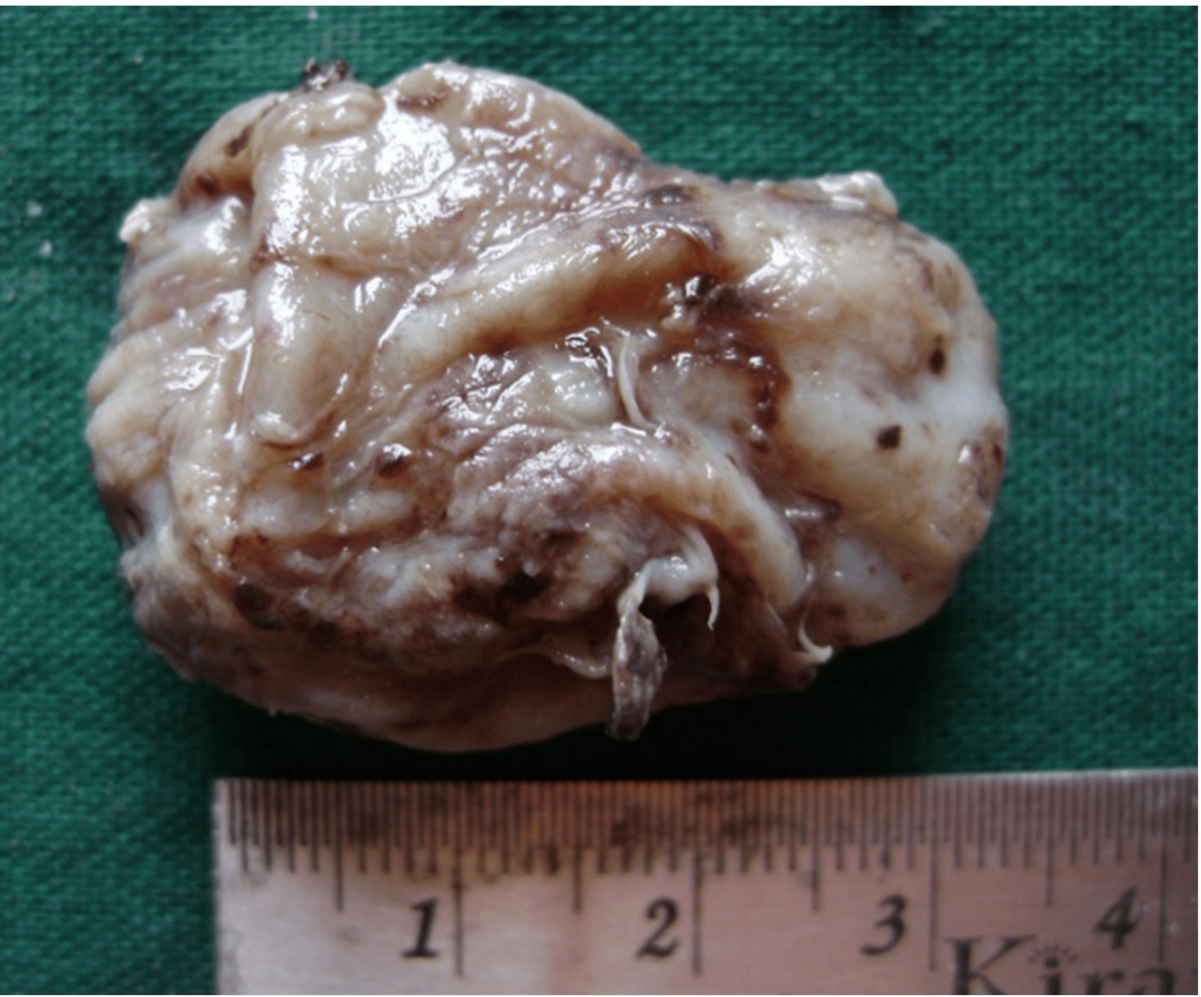
Excised ovoid tumour mass

Considering the clinical location and radiographic findings, a provisional diagnosis of AOT was made. Differential diagnosis included ameoblastoma and odontogenic keratocyst because the lesions can exhibit a similar clinical behavior and radiographic appearance. The lesion was completely excised under local anaesthesia and the specimen was sent as an excisional biopsy for histopathological evaluation. Grossly, the specimen appeared cystic, ovoid in shape, and cream to brownish in colour, measuring approximately 4 × 2.5 cm (Figure 3).

The internal cavity of the cyst was empty, except for small blood clots and debris. The cystic wall thickness varied from 0.5 mm to 2 mm.

Histopathological examination

**Figure 4:**
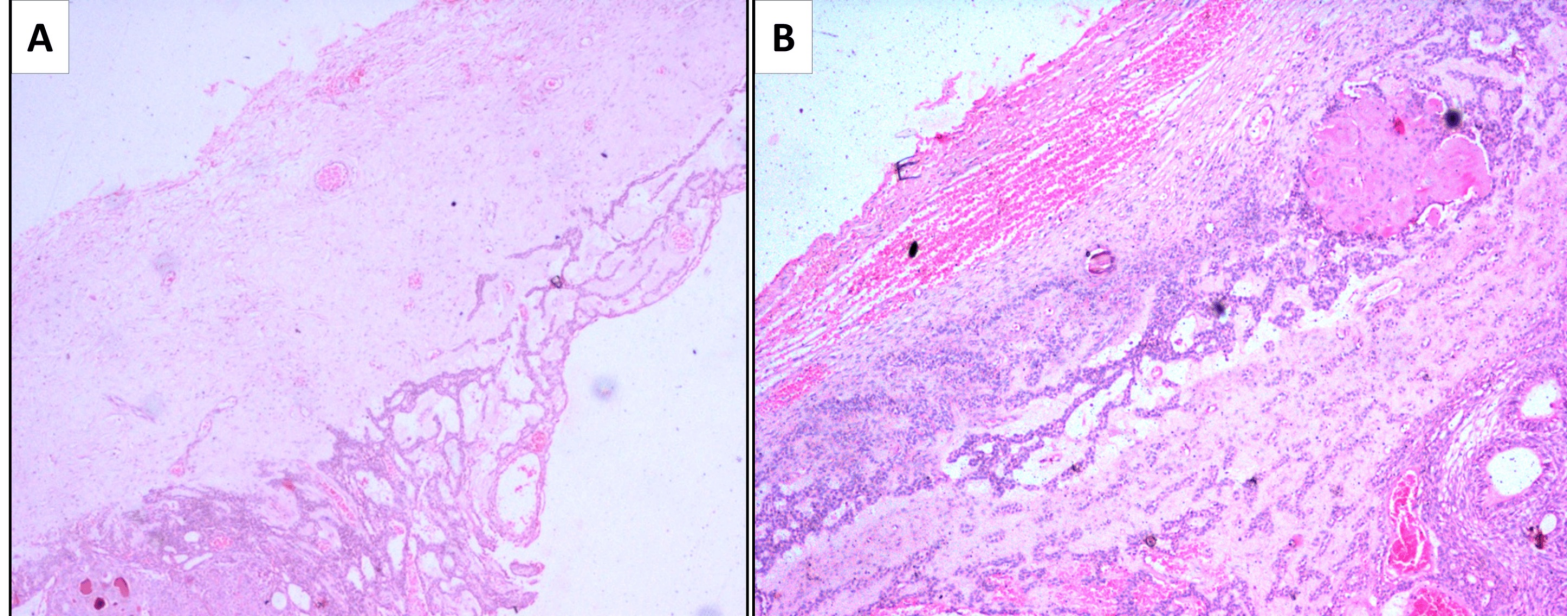
Hemotoxylin and Eosin (H &E) staining A) Scanner view showing cystic lumen lined by cystic lining and tumor nodules (H & E stain, 4x magnification); B) Low power view exhibiting cuboidal to spindle-shaped cells arranged in whorls, ducts, sheets, and cords (H & E stain, 10x magnification)

Microscopic analysis of hematoxylin and eosin-stained sections revealed a non-keratinized, stratified squamous odontogenic epithelium lining of two to three cell layers, exhibiting focal areas of proliferation, lining a cystic lumen. Within these areas, the epithelial cells formed sheets, cords, and characteristic whorls and duct-like structures composed of ovoid to spindle-shaped odontogenic cells (Figure 4).

Some of the ductal structures exhibited a hyaline eosinophilic ring within their lumen.

**Figure 5:**
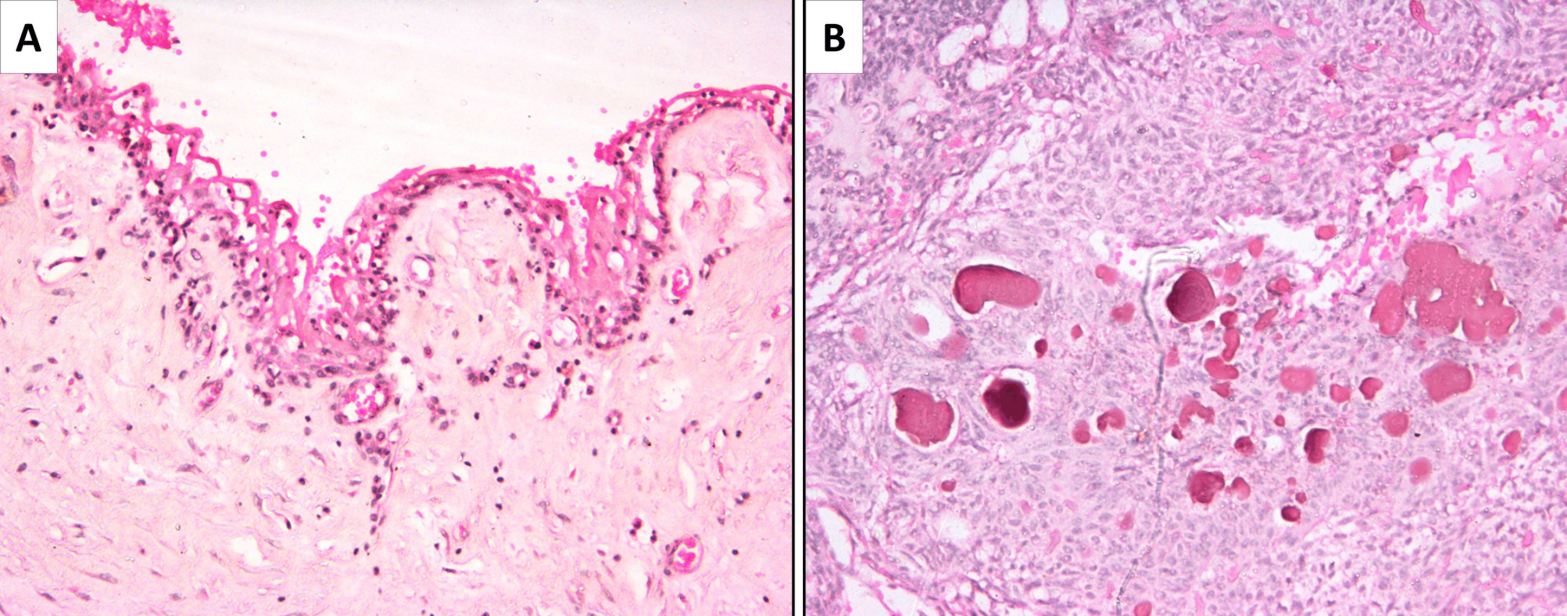
Hematoxylin and Eosin (H & E) staining of the stroma A) Cystic lumen lined by two to three layers of odontogenic epithelium; B) Large areas of eosinophilic secretary material and focal collections of round, basophilic calcifications with eosinophilic droplets. (H & E stain, 10x magnification).

The highly cellular connective tissue stroma enclosed eosinophilic secretory material and displayed small focal calcifications scattered within the areas of stroma (Figure 5).

Areas of squamous metaplasia with keratin formation were also observed, which represented metaplastic changes in the odontogenic epithelium. The connective tissue stroma underlying the epithelial proliferation appeared highly vascular, edematous, and focally hyalinized. Considering the histopathological features and correlation with clinical and radiographic findings, a definitive diagnosis of the extrafollicular variant of AOT was established.

## Discussion

AOT is a benign, hamartomatous epithelial lesion accounting for approximately 2.2% to 13% of all odontogenic tumours [6]. It predominantly affects young female patients in the second decade of life, with a strong predilection for the anterior maxilla [2,6,7]. The current case conforms to these epidemiological trends, involving a 23-year-old female patient with a lesion in the anterior maxilla. A review of cases of extrafollicular AOT published in the literature is summarized in Table 1.

**Table 1:** Cases of extrafollicular adenomatoid odontogenic tumor published in recent scientific literature CBCT: Cone Beam Computed Tomography; CT: Computed Tomography; AOT: Adenomatoid odontogenic tumour; RCT: Root Canal Treatment.

Author (Year)	Age/Sex	Location	Clinical features	Radiographic features	Association with the tooth	Root resorption	Histopathological features	Treatment	Follow-up
Kundoor et al. (2016) [8]	20/F	Anterior maxilla	Swelling in the upper left anterior region for 5 to 6 months; hard, non-tender; vestibular obliteration	Well-defined unilocular radiolucency with discrete calcifications; located interradicularly between maxillary left lateral incisor and first premolar	No	No	Duct-like structures, rosette-like epithelial cells, eosinophilic material and calcifications	Conservative surgical enucleation	No recurrence at three months
Singla et al. (2018), Case 1 [2]	14/F	Anterior mandible	Incidental finding; mild labial expansion; spacing between lower lateral incisor & canine	Well-defined 2-3 cm unilocular radiolucency; interradicular between right lateral incisor and left canine; displacement of canine root	No	No	Duct-like structures and epithelial sheets	Surgical enucleation	Not mentioned
Singla et al. (2018), Case 2 [2]	17/M	Anterior maxilla (left)	Swelling post trauma; firm, vestibular obliteration; mesial drift of canine	Corticated radiolucent lesion with specks of calcification on occlusal view; not associated with an unerupted tooth	No	No	Duct-like structures, spindle and cuboidal epithelial cells, columnar epithelium with polarized nuclei	Surgical excision	Not mentioned
Mosavat et al. (2018) [9]	40/F	Anterior mandible	Asymptomatic; incidental finding, bony swelling	Unilocular radiolucency, CBCT showed internal calcifications, root deviation	None	Yes, lateral incisor and canine	Spindle-shaped cells in sheets, duct-like and rosette structures; small calcifications, infiltration of capsule	Enucleation	Not mentioned
De et al. (2020) [10]	13/M	Right mandible (canine and premolar region)	Swelling for six months; intraoral exophytic gingival mass; displacement of right lateral incisor and right canine	Unilocular well-defined radiolucency with radiopaque flecks; displacement of canine; buccal and lingual cortical expansion; extends from lateral incisor to second premolar	No impacted tooth; extrafollicular type	Not reported	Cuboidal to columnar cells in nests, rosettes, whorls, duct-like structures, fibrous stroma with blood vessels and RBCs	Surgical excision	Not mentioned
Costa et al. (2022) [11]	16/M	Anterior maxilla (between right central and lateral incisors)	Incidental finding; mild buccal swelling; asymptomatic	Well-defined hypodense lesion with hyperdense foci; interradicular; buccal cortical expansion and erosion	No association with impacted tooth; extrafollicular (interradicular)	Not reported	Typical AOT features (duct-like and rosette structures)	Excisional biopsy with curettage	Not mentioned
Deb et al. (2022) [12]	20/F	Right maxilla (anterior region)	Slow-growing swelling over eight months, intraoral vestibular obliteration, mobility of right canine	Unilocular radiolucency with thin corticated border; buccal cortical expansion and calcific specks	No impacted tooth; extrafollicular type involving right canine	Not mentioned	Rosette and duct-like odontogenic epithelial cells, fibrous capsule, amyloid-like material	Surgical excision with involved tooth	Not mentioned
Sharma et al. (2022) [13]	33/M	Left maxillary anterior region	Painless swelling for one to two months; intraoral 2 X 2 cm lesion; incomplete root canal therapy previously done	Large unilocular radiolucency with well-defined border from left central incisor to first premolar; slight root resorption	Not associated with impacted tooth; extrafollicular (between erupted teeth)	Slight root resorption noted	Rosettes, cords, duct-like structures with eosinophilic material in lumen	Surgical enucleation and curettage	Not mentioned
Rabanales et al. (2023) [6]	15/F	Anterior mandible (left)	Asymptomatic swelling in parasymphyseal region; firm; ~4 X 4 cm	Unilocular radiolucency encompassing erupted left mandibular canine; CT: cortical expansion and perforation; no root resorption	Yes (erupted tooth)	No	Islands and pseudo-ducts of odontogenic epithelium; dystrophic calcifications; well-vascularized fibrous stroma	Enucleation and curettage	No recurrence at 14 months
Vichattu et al. (2023) [14]	22/F	Anterior maxilla (right)	Swelling, facial asymmetry, nasal ala elevation, vestibular obliteration, mobility of adjacent teeth	Unilocular radiolucency with calcifications; buccal cortical expansion and perforation; root deviation; sinus floor elevation	No impacted tooth; interradicular extrafollicular type	Yes (central incisor and first premolar)	Duct-like, rosette, and whorled epithelial patterns with calcifications	Surgical enucleation; palatal splinting; RCT of affected teeth	Not mentioned
Osundwa et al. (2025) [15]	16/F	Right anterior mandible (parasymphyseal region)	Painless swelling for eight months, buccolingual expansion, tooth displacement	Well-defined unilocular radiolucency with sclerotic border, displacing the mandibular right first premolar.	Extrafollicular (associated with erupted right first premolar)	Partial root engulfment of right first premolar	Well-encapsulated, duct-like and rosette structures, eosinophilic material, minimal atypia	Surgical enucleation	Not mentioned
Present case (2025)	23/F	Anterior maxilla (left)	Painless swelling, slow growth, buccal & palatal expansion	Well-defined unilocular radiolucency with corticated margins; from maxillary central incisor to first molar; displacement of canine; nasal septum deviation	No	Yes (central incisor, lateral incisor, second premolar)	Duct-like structures, rosettes, eosinophilic material, calcifications, squamous metaplasia, hyalinized stroma	Surgical excision (enucleation)	No recurrence at two years

Traditionally, AOT is known for its “two-thirds” pattern: two-thirds of cases arise in females, two-thirds in the maxilla, and two-thirds are associated with unerupted teeth, commonly canines [1,3]. However, our case deviated from the classical follicular pattern, instead presenting as an extrafollicular variant that is defined by its lack of association with an impacted tooth [7]. Extrafollicular AOT accounts for approximately 24% of cases and may occur in periapical, interradicular, or superimposed locations [7]. In the present case, the lesion was interradicular and showed no direct relationship to any unerupted tooth, thereby fitting the extrafollicular subtype.

The extrafollicular AOT can be subclassified as E1 (no relation to tooth structure), E2 (interradicular with divergence of adjacent roots), E3 (superimposed on root apex), or E4 (superimposed on mid-root) [2,6,8,9,16]. The lesion reported here appears to resemble the E2 pattern, as it was located interradicularly with signs of mesial displacement of the left maxillary canine and root resorption of the adjacent central incisor, lateral incisor, and second premolar. Root resorption, although uncommon in AOT, has been reported in select cases, such as those by Mosavat et al. and Vichattu et al., and was similarly noted in our patient [9,14]. In the present case, root resorption observed in the maxillary central incisor, lateral incisor, and second premolar was most likely pressure-induced, resulting from the expansile nature and size of the lesion, which extended across the interradicular space and displaced adjacent structures. The lesion’s intact corticated borders, lack of association with non-vital or previously extracted teeth, and displacement of adjacent teeth rather than apical ballooning favoured a diagnosis other than a periapical or residual cyst. Furthermore, the lesion’s size and extension beyond a single tooth region, combined with the absence of periapical pathology in pulp vitality testing, supported its non-cystic neoplastic nature.

Radiographically, AOT usually presents as a well-demarcated unilocular radiolucency. In the follicular variant, it is typically associated with the crown of an impacted tooth, while the extrafollicular form often mimics other periapical pathologies [2,6,9]. Because extrafollicular AOTs are not associated with impacted teeth and often present between or near the roots of erupted teeth, they can radiographically resemble periapical cysts, granulomas, or other inflammatory odontogenic lesions, leading to frequent misdiagnoses. In the present case, the lesion extended from the mesial surface of the left maxillary central incisor to the mesial surface of the left maxillary first molar, causing considerable displacement of the adjacent canine and deviating the nasal septum. These features could easily lead to a misdiagnosis of a radicular cyst or other odontogenic cysts.

Extrafollicular AOT may resemble a range of radiolucent lesions in the anterior maxilla, including periapical cysts, residual cysts, and central giant cell granulomas. However, AOT typically presents as a well-defined, corticated unilocular radiolucency that may cause displacement of adjacent teeth without significant root resorption, although exceptions exist [12]. Unlike periapical cysts, AOT lesions are vital-tooth-associated and generally non-inflammatory. Residual cysts, which occur at previously extracted tooth sites, tend to lack cortical expansion and calcific foci [9]. Central giant cell granulomas often show a multilocular pattern with ill-defined margins and may cause bone perforation or root divergence rather than displacement [15].

Histologically, AOT is distinctive due to its duct-like structures, rosette formations, and spindle or polygonal epithelial cells in sheets, cords, or whorls, often embedded in a loosely collagenous connective tissue stroma. It frequently contains calcified material and eosinophilic secretory deposits [1,2]. Although histopathology revealed prominent foci of calcification, they were not extensive enough to produce discernible radiopacities on conventional imaging in this case, consistent with previous reports where fine calcifications were radiographically inapparent [8,9]. Our case exhibited all hallmark features, including proliferating odontogenic epithelium arranged in nodules, whorls, and duct-like structures filled with eosinophilic secretory material and foci of calcifications. Additionally, squamous metaplasia with keratinization and a highly vascular, hyalinized stroma were observed. These findings are consistent with those reported by Reichart et al. and are typical of the AOT histological spectrum [1].

Interestingly, some studies have identified the presence of dysplastic dentinoid-like material within AOT, considered to be metaplastic rather than true dentin [1,3]. In rare instances, areas resembling calcifying epithelial odontogenic tumour or ameloblastoma may also be present, suggesting histological overlap within benign odontogenic tumours [17]. Although our case did not show such hybrid features, the presence of keratinization and extensive calcifications suggests a broad histological spectrum that may mimic other odontogenic entities. Squamous metaplasia, although not a classical feature of AOT, has occasionally been reported in both follicular and extrafollicular variants. Its presence in our case likely reflects secondary changes due to chronic irritation or lesion maturation, rather than a defining histological hallmark. The presence of squamous metaplasia and keratinization in the current case did raise initial histopathological considerations of other keratin-producing odontogenic lesions, such as odontogenic keratocyst or a solid variant of calcifying odontogenic cyst. However, the overall architecture, duct-like structures, rosette patterns, and eosinophilic secretory material remained consistent with AOT, thus ruling out these mimics.

Immunohistochemically, AOT demonstrates low proliferative activity and benign biological behavior, with markers such as p53 and Proliferating Cell Nuclear Antigen (PCNA) expressed at significantly lower levels than in ameloblastoma, reinforcing its non-aggressive nature [18]. The well-encapsulated structure of AOT further supports the effectiveness of conservative management. As in previous reports, surgical enucleation remains the treatment of choice, with excellent prognosis and minimal risk of recurrence [8,19]. In the present case, complete surgical excision was performed, and no recurrence was observed during a two-year follow-up period.

Although the clinical presentation of a slow-growing, painless swelling in a young female patient involving the anterior maxilla is suggestive of a benign odontogenic lesion, these features alone are not specific for extrafollicular AOT. Early suspicion may arise when the lesion demonstrates asymptomatic expansion without signs of inflammation and lacks association with non-vital teeth, but definitive diagnosis requires radiographic correlation and histopathologic confirmation. Although recurrence of AOT is exceptionally rare due to its benign and encapsulated nature, a structured follow-up protocol involving clinical and radiographic assessments at six-month intervals for the first year, followed by annual reviews for at least two to three years, is advisable particularly for extrafollicular variants, where atypical behavior or misdiagnosis may delay treatment. No recurrence was noted in our case over a two-year follow-up period.

## Conclusions

The present case report underscores the importance of considering the extrafollicular variant of AOT in the differential diagnosis of anterior maxillary radiolucencies, particularly when not associated with an unerupted tooth. The clinical presentation of a swelling along with radiographic features, including root resorption and displacement of adjacent teeth, and characteristic histopathological architecture, enabled a definitive diagnosis. Although rare, extrafollicular AOT should not be overlooked, especially in young female patients presenting with slowly enlarging, well-circumscribed maxillary lesions. Timely recognition and conservative surgical management remain crucial, as AOT is benign, well-encapsulated, and associated with an excellent prognosis when completely excised.
